# Lexical quality and executive control predict children’s first and second language reading comprehension

**DOI:** 10.1007/s11145-017-9791-8

**Published:** 2017-11-02

**Authors:** Henriette Raudszus, Eliane Segers, Ludo Verhoeven

**Affiliations:** Behavioural Science Institute, Radboud University, Montessorilaan 3, P.P. Box 9104, 6500 HE Nijmegen, The Netherlands

**Keywords:** Reading comprehension, Bilingualism, Lexical quality, Executive control, Syntactic integration

## Abstract

This study compared how lexical quality (vocabulary and decoding) and executive control (working memory and inhibition) predict reading comprehension directly as well as indirectly, via syntactic integration, in monolingual and bilingual fourth grade children. The participants were 76 monolingual and 102 bilingual children (mean age 10 years, *SD* = 5 months) learning to read Dutch in the Netherlands. Bilingual children showed lower Dutch vocabulary, syntactic integration and reading comprehension skills, but better decoding skills than their monolingual peers. There were no differences in working memory or inhibition. Multigroup path analysis showed relatively invariant connections between predictors and reading comprehension for monolingual and bilingual readers. For both groups, there was a direct effect of lexical quality on reading comprehension. In addition, lexical quality and executive control indirectly influenced reading comprehension via syntactic integration. The groups differed in that inhibition more strongly predicted syntactic integration for bilingual than for monolingual children. For a subgroup of bilingual children, for whom home language vocabulary data were available (*n* = 56), there was an additional positive effect of home language vocabulary on second language reading comprehension. Together, the results suggest that similar processes underlie reading comprehension in first and second language readers, but that syntactic integration requires more executive control in second language reading. Moreover, bilingual readers additionally benefit from first language vocabulary to arrive at second language reading comprehension.

## Introduction

A significant number of children around the world learn to read in a language other than their native tongue. Previous research has shown that while these bilingual readers acquire adequate decoding skills, they show poorer reading comprehension than their monolingual peers (Melby-Lervåg & Lervåg, 2014). In a literate society, reading comprehension problems are a major obstacle for social inclusion and academic success. It is therefore important to understand what causes bilingual readers’ weaker comprehension. Previous research has clearly shown that lower second language (L2) vocabulary plays a role in L2 reading comprehension problems (e.g., Burgoyne, Kelly, Whiteley, & Spooner, 2009; Lervåg & Aukrust, 2010). This concerns both the number and the quality of word representations (Proctor, Silverman, Harring, & Montecillo, 2012). Prominent models of comprehension posit that this lexical quality is important because it serves as input to integration processes at the sentence level (Hagoort, 2013; Perfetti & Stafura, 2014). However, this syntactic integration is under-investigated for L2 reading comprehension. Combining words into larger units of representation also relies on executive control, such as working memory and inhibition (Hagoort, 2005). Executive functions have been shown to impact first language (L1) reading (e.g., Cain, 2006), but little is known about their role in L2 reading comprehension. It has been suggested that L2 readers rely on these control functions to a larger extent than monolinguals to compensate for their weaker target language proficiency (Prehn, Taud, Reifegerste, Clahsen, & Flöel, 2017). Furthermore, bilinguals could also profit from their knowledge of L1 in order to arrive at L2 comprehension (Cummins, 1989). Studies on the role of L1 in L2 reading comprehension are sparse and show mixed results (e.g., Proctor, August, Carlo, & Snow, 2006; Proctor et al., 2012). To arrive at a better understanding of the components involved in first and second language reading comprehension the current study investigated how lexical quality and executive control contribute to reading comprehension via syntactic integration, and whether bilingual readers can profit from L1 vocabulary in addition to L2 and cognitive predictors.

A seminal model of reading comprehension is the Reading Systems Framework (Perfetti & Stafura, 2014), which states that word-to-text integration is central to comprehension. In this model, orthographic and phonological information from word decoding is used to access word meaning. Quick and accurate retrieval is made possible by high-quality lexical representations. These contain detailed information on orthographic, phonological, and semantic characteristics (Perfetti, 2007; Perfetti & Hart, 2002). The recognized words are then integrated into the surrounding context: Syntactic integration leads to a representation of the sentence, which in turn enables text comprehension. The central role of local integration processes in language comprehension is also posited by the Memory, Unification, and Control framework (Hagoort, 2005, 2013). This framework additionally emphasizes the role of control processes such as working memory and inhibition. These control processes are necessary to guide the unification of elements retrieved from the mental lexicon into larger units with new meaning. For example, as a reader recognizes successive words in a sentence, these words have to be held in working memory until grammatical or semantic ambiguities are resolved by inhibition of the least plausible interpretation. In the following, we will discuss how lexical quality, syntactic integration, and executive control influence L1 and L2 reading comprehension.

Regarding lexical quality, it is well known that vocabulary and decoding predict reading comprehension in monolingual as well as bilingual readers (Melvy-Lervåg & Lervåg, 2014). More specifically, the number of words known (vocabulary breadth), as well as the quality of their representation (vocabulary depth), are important. For example, Richter, Isenberner, Naumann, and Neeb (2013) showed that the quality of orthographic, phonological, and semantic information, as well as the efficiency of access to this information, contribute to reading comprehension in grades 1–4. In a study with fifth-grade L1 and L2 readers of Dutch, Cremer and Schoonen (2013) found that L2 readers’ lower reading comprehension was explained by lower accessibility of semantic knowledge, while monolinguals and bilinguals did not differ on word decoding. This is in line with a growing literature suggesting that for bilingual readers, decoding does not present a particular problem, but that the limited number and quality of L2 lexical representations is what hinders L2 comprehension (e.g., Babayiğit, 2014; Burgoyne et al., 2009; Lervåg & Aukrust, 2010). Good decoding in the absence of vocabulary knowledge is the key to an empty vault—it will not aid comprehension.

There are indications that for bilingual readers, target language vocabulary might be even more important than for monolinguals. For example, Droop and Verhoeven (2003) reported that for Turkish–Dutch and Moroccan–Dutch children in the intermediate primary grades, Dutch language skills were more strongly related to Dutch reading comprehension than for Dutch monolingual children. Similar results were found by Lervåg and Aukrust (2010) for Urdu-Norwegian bilinguals in the lower primary grades, and by Babayiğit (2014) for L2 English readers with diverse language backgrounds in fifth grade. This was not replicated, however, in a later study by Babayiğit (2015), in which no structural differences between monolingual and bilingual readers were found. Similarly, Bowyer-Crane et al. (2016) found no differences in the predictors of L1 and L2 reading comprehension when they compared second-grade bilingual children to monolingual children with language weaknesses. It remains to be clarified whether and under what circumstances target language vocabulary is more important for L2 than for L1 reading comprehension.

Lexical quality is crucial because it is the basis for higher-level processing: Word-level representations feed into the unification system to arrive at syntactic integration, which in turn is the basis for text understanding. Well-defined and speedily accessible lexical representations facilitate this process. While the role of lexical quality in text-level reading comprehension has been extensively investigated, syntactic integration processes have received far less attention. There is some evidence that poor reading comprehension skill is associated with syntactic difficulties in monolinguals (Mokhtari & Thompson, 2006; Muter, Hulme, Snowling, & Stevenson, 2004; Nation & Snowling, 2000), but research on the role of syntactic integration in L2 reading comprehension is sparse. When a measure of syntax is included in L2 research, it is often subsumed under a general language component (e.g., Babayiğit, 2015; Bowyer-Crane et al., 2016), or vocabulary is not controlled for in addition to syntax (e.g., Lesaux, Lipka, & Siegel, 2006). Proctor et al. (2012) did investigate the unique contribution of syntax and found that syntax predicted English reading comprehension in monolingual English and Spanish–English bilinguals in second to fourth grade, after controlling for vocabulary breadth. No interaction between language background and syntax was found. Geva and Farnia (2012) compared monolingual Canadian English and bilingual children with diverse language backgrounds, and found that in fifth grade, English syntax predicted English reading comprehension on top of nonverbal ability, decoding, and vocabulary for L2 but not L1 readers. Adding to this, Farnia and Geva (2013) reported that grade 1 syntax is the most important positive predictor of reading comprehension rate of growth in both L1 and L2 readers. Hence, the role of syntactic integration in bilingual as compared to monolingual reading comprehension warrants further investigation.

Syntactic integration relies on vocabulary, but it also requires control: Several elements have to be held in working memory until integration has taken place. In addition, conflicts in integration have to be resolved through inhibition (Hagoort, 2005). It has been suggested that holding information in mind and manipulating it as more information from the text is processed could be a central weakness in monolingual readers with comprehension problems (Cain, 2006; Sesma, Mahone, Levine, Eason, & Cutting, 2009). Not much is known about executive control in L2 reading comprehension, but there is no reason to assume that poor executive control would be a weakness in bilinguals. On the contrary, it has been suggested that, in general, bilingualism is associated with improved inhibition, brought about by handling two languages and suppressing the non-used language continuously (e.g., Bialystok, 2009). Recently, this claim has been disputed (e.g., De Bruin, Treccani, Della Sala, 2015; Paap & Sawi, 2014), but even if bilinguals show normal inhibition—as opposed to improved control—it is conceivable that they can use this to their advantage to compensate for poorer target language proficiency (Prehn et al., 2017; Sebastian, Laird, & Kiran, 2011).

In all, it is clear that bilinguals differ from monolinguals in their target language proficiency, and possibly their executive control skills. An additional difference is that, by definition, they also know another language, their L1, but most studies on reading comprehension only report competence in the L2. It is, however, well recognized that L1 and L2 lexicons are not separate entities, but closely intertwined (for a review, see De Groot, 2011). It is therefore possible that a well-developed L1 vocabulary benefits L2 reading comprehension, as L1 and L2 vocabulary both contribute to richness of the reader’s conceptual network (Cummins, 1989). There are a few studies suggesting that L1 proficiency does, indeed, play a role in L2 reading comprehension. For instance, Manis, Lindsey, and Bailey (2004) found that L1 Spanish language skills in kindergarten explained a small amount of variance in L2 English reading in the second grade, but that this contribution was mediated by English language proficiency. Proctor et al. (2006) found that L1 Spanish proficiency explained a small but significant amount of variance in L2 reading comprehension after controlling for L2 competence. Lervåg and Aukrust (2010) reported similar results for Norwegian-Urdu bilinguals. It is thus important to take into account L1 for a more complete picture of predictors of L2 reading comprehension (but see Proctor et al., 2012).

Taken together, reading comprehension is an important skill in which bilingual students lag behind their monolingual peers. From previous research, it is clear that weaker target language vocabulary plays a role in this. Furthermore, there are indications that target language vocabulary might be even more important in L2 than in L1 reading comprehension. Syntactic integration is likely to be a stepping stone between vocabulary and reading comprehension, and this central link is under-investigated. How lexical quality and executive control act in concert to arrive at syntactic integration in monolingual and bilingual reading comprehension deserves further attention. We suggest a model in which lexical quality (vocabulary and decoding) and executive control (working memory and inhibition) substantially contribute to reading comprehension directly as well as indirectly via syntactic integration. We investigated this model in a sample of monolingual and bilingual children in fourth grade in the Netherlands, with the aim to (1) compare the levels of lexical quality, executive control, syntactic integration and reading comprehension in monolinguals and bilinguals, (2) examine to what extent the predictors of reading comprehension differ between L1 and L2 readers, and (3) explore whether L1 vocabulary influences L2 reading comprehension in addition to L2 and cognitive predictors.

## Method

### Participants

Participants in this study were 178 fourth grade children (90 girls, 88 boys), with an average age of 10 years (*SD* = 5 months), of which 102 (57%) were bilingual. The children came from ten fourth grade classrooms in seven schools in cities in the south of the Netherlands. In the participating schools, all children took part in the study, unless their parents or guardians declined participation in a passive consent procedure. Children with a diagnosis of dyslexia were also excluded from participation, as were children who were born outside of the Netherlands, as it can be assumed their exposure to Dutch was different from the other participants. The bilingual children’s language background was diverse. The largest groups consisted of speakers of Turkish (21%), Moroccan Arabic (19%) and Berber (16%).

Bilingualism status and amount of home language use were investigated by means of an oral questionnaire. All children were asked if they spoke any language other than Dutch. If they answered yes, they were asked to indicate what language they spoke with their mother, father, siblings, friends, and extended family. For each of those questions, the answer was categorized as 1 “only Dutch”, 2 “mostly Dutch, sometimes other language”, 3 “mostly the other language, sometimes Dutch”, or 4 “only the other language”. All children with a score higher than 1 on any of the questions were coded as bilingual in subsequent analyses (following e.g. Babayiğit, 2014; Cremer & Schoonen, 2013; Melby-Lervåg & Lervåg, 2014). Of the bilingual children, the largest group reported to speak mostly their home language with their parents and extended family, and mostly or only Dutch with siblings and friends. These language usage patterns are consistent with earlier reports from the literature (e.g., Lervåg & Aukrust, 2010; Unsworth, 2016).

Monolinguals and bilinguals did not differ significantly in age in months (*M*_L1_ = 119.4, *SD*_L1_ = 5.2; *M*_L2_ = 119.9, *SD*_L2_ = 5.3; *t*(176) = − .64, *p* = .53, *d* = − .10) or nonverbal reasoning ability as measured by Raven’s Standard Progressive Matrices (Raven, 2006) (*M*_L1_ = 39.9, *SD*_L1_ = 7.2; *M*_L2_ = 37.9, *SD*_L2_ = 5.3; *t*(167) = 1.85, *p* = .07, *d* = .29).

### Materials

#### Vocabulary

Vocabulary depth in Dutch was measured by the word definition subtask of the Taaltoets Allochtone Kinderen Bovenbouw [Language Test for Minority Children Grades 4–6] (Verhoeven & Vermeer, 1993). In this task, the experimenter named a word, which the child was asked to define. There were 25 items, and the definitions were scored 2 points for a formal definition, 1 point for a functional description, and 0 points for an incorrect or missing response. A higher score indicates larger vocabulary depth. Cronbach’s α for this test is .90 (Verhoeven & Vermeer, 1996).

Vocabulary breadth in Dutch was measured by the Dutch Peabody Picture Vocabulary Test-III (PPVT-III-NL; Schlichting, 2005). For this task, children were presented with four pictures. The experimenter said a word, and the child had to point to the picture best depicting the word. Items were grouped in sets of 12. Assessment was discontinued after 9 errors in one set of 12. Reliability (lambda2-coefficient) ranges between .96 and .95 for children between 9 and 11 years old (Schlichting, 2005).

For the multigroup analyses, Dutch vocabulary depth and breadth were combined into one ‘vocabulary’ score by standardizing and summing the respective scores.

Vocabulary breadth in the home languages for children who spoke Turkish, Moroccan Arabic or Berber was tested with a similar paradigm as vocabulary breadth in Dutch, using the Passive Vocabulary subtest of the Toets Tweetaligheid [Diagnostic Test of Bilingualism] (Verhoeven, Narain, Extra, Konak, & Zerrouk, 1995). The test was available in Turkish and Moroccan Arabic and was translated to Berber (Nador dialect) by a native speaker of Moroccan Arabic and Berber. The test consisted of 60 items. The child listened to a recording of a native speaker of their language saying a word, and had to point to the corresponding picture. Each correct choice of picture was awarded one point. Cronbach’s α for this test is .90 (Verhoeven et al., 1995).

#### Decoding

The children’s word decoding skill was measured by the Een-Minuut-Test [One Minute Test] Version B (Brus & Voeten, 1999). For this task, children read as many words of increasing length as possible within 1 min, with the difficulty of words increasing from consonant–vowel–consonant to multisyllabic words with consonant clusters. Each word read correctly was awarded one point, with a maximum of 116. Parallel test reliability ranges from .89 to .92 (Brus & Voeten, 1999).

Pseudoword decoding skill was measured by the Klepel Test Version B (Van den Bos, Lutje Spelberg, Scheepstra, & de Vries, 1994). For this test, children read as many pseudowords of increasing difficulty as possible within 2 min. Each item read correctly was awarded one point, with a maximum of 116. Parallel test reliability is .89 (Van den Bos et al., 1994).

For the multigroup analyses, word and pseudoword decoding were combined into one ‘decoding’ score by standardizing and summing the respective scores.

#### Syntactic integration

Syntactic integration was assessed by the grammar judgement task of the Taaltoets Allochtone Kinderen Bovenbouw [Language Test for Minority Children Grades 4–6] (Verhoeven & Vermeer, 1993). Children were presented with sets of three sentences and had to indicate which one was wrong, or whether they thought all were correct. The target sentences in the task contained morphological and syntactic errors concerning difficult features of Dutch grammar, such as gender agreement, case marking on pronouns, and order of sentence constituents. There were 40 items and each correct answer was awarded one point. Cronbach’s α for this test is .86 (Verhoeven & Vermeer, 1996).

#### Working memory

Working memory was assessed by the backward digit span subtask of the Wechsler Intelligence Scale for Children III, Dutch version (WISC-III-NL; Kort et al., 2005). In this task, the experimenter named digits at a rate of approximately one per second. The child repeated the digits in the reverse order. Every two trials, the number of digits increased by one, until the child failed to repeat two trials of the same length correctly. Every trial that was repeated correctly was scored as one point, with a maximum score of 16. Cronbach’s α and split-half reliability indices range from .50 to .66 (Kort et al., 2005).

#### Inhibition

Inhibition was assessed by means of the Simon task (Simon & Wolf, 1963) implemented in PsychoPy (version 1.84.1; Peirce, 2007). In this task, children were presented with one of two different pictures on a screen. They were instructed to always press the left button on a button box when they saw one picture, and the right button when they saw the other picture. On congruent trials, the stimulus appeared on the side of the button that had to be pressed. On incongruent trials, the stimulus appeared on the opposite site, meaning that irrelevant location information had to be suppressed. In our implementation of the Simon task, stimuli were a butterfly (left button) and a frog (right button), and half of the trials were incongruent. Children could practice for eight trials. The task then proceeded with 80 experimental trials. Each trial started with a fixation cross in the middle of the screen for 1.5 s, after which the stimulus appeared until a button was pressed. There was no feedback or timeout. Four participants with accuracy lower than 50% on both the congruent and the incongruent condition were excluded from the reaction time analysis, as we assume they misunderstood the instructions or were not capable of performing the task. For the remaining participants, only trials on which the correct button was pressed (91.2%) were included in the reaction time analysis. The median reaction time in milliseconds was calculated by condition for each participant. The difference between median incongruent and congruent trial reaction time was taken as a measure of inhibition. We mirrored the difference score such that a smaller difference between conditions is associated with a larger score, indicating better inhibition.

#### Reading comprehension

Reading comprehension was assessed by the Text Reading Task of the Taaltoets Allochtone Kinderen Bovenbouw [Language Test for Minority Children Grades 4–6] (Verhoeven & Vermeer, 1993). Children read two texts of approximately 300 words with 20 gaps each. For each gap, children chose which of three words fit into the gap best. To choose correctly, information from several sentences and world knowledge had to be considered. Each gap filled correctly was awarded one point, resulting in a maximum of 40 points. Cronbach’s α for this test is .75 (Verhoeven & Vermeer, 1996).

### Procedure

Approval for this study was obtained from the Ethics Committee of the Faculty of Social Sciences at Radboud University. Schools with a high population of bilingual students were contacted by phone by the first author, and those schools interested in participating were included in the sample. Schools received information about the findings of the study and individual results in exchange for their collaboration. The children’s parents or guardians were informed of the project via letter, and could indicate if they did not want their child to take part in the study.

Children were tested in the second half of fourth grade. Tests were administered by the first author and seven trained undergraduate students of Educational Science. The syntactic integration and reading comprehension tests were administered in one classroom session. Each of the tests took 30 min, and there was a 15–20-min break in between. In a second classroom session, children were allowed 40 min to complete the nonverbal reasoning task. All other tasks were completed in two individual sessions lasting 30–45 min each. The individual sessions also included computerized mind-mapping, self-paced reading and inferencing tasks used for another study.

### Analyses

**Fig. 1:**
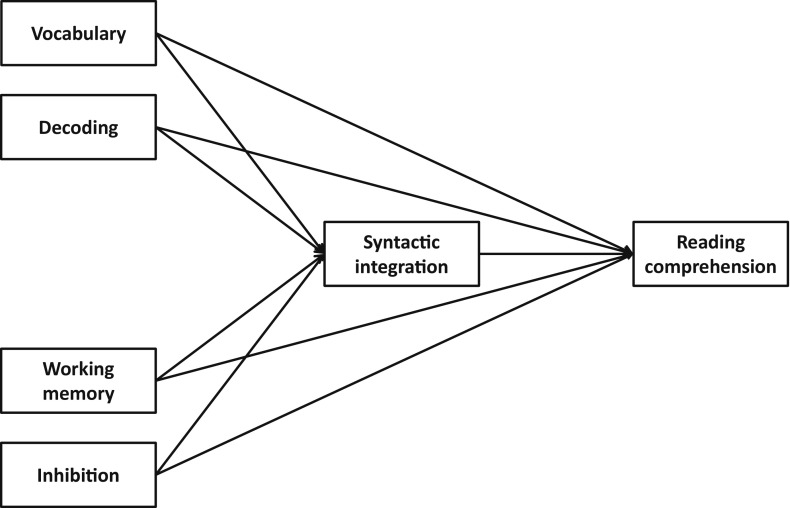
Hypothesized model of predictors of reading comprehension

Whether L1 and L2 children differed in mean performance on the predictors of reading comprehension (RQ 1) was assessed by means of two-tailed *t* tests. To investigate possible differences in regression coefficients between the predictors of L1 and L2 reading comprehension (RQ 2), multigroup path analyses were carried out. Our initial theoretical model included direct paths from vocabulary, decoding, working memory, and inhibition to reading comprehension as well as indirect paths of those variables via syntactic integration (see Fig. 1). Exogenous variables were allowed to covary. To test for group differences in regression coefficients, we first computed a model in which all regression coefficients were allowed to vary between groups. Then, for each regression path we tested whether constraining the coefficient to be equal for the L1 and the L2 group significantly decreased model fit. If model fit did not decrease, the constraint was retained in the final model, as a more constrained model is more parsimonious. If the constraint did decrease model fit, the parameter in question was freely estimated in the final model.

To investigate the role of L1 vocabulary in L2 reading comprehension (RQ3), the final model from the procedure described above was fitted for the group of L2 children for whom L1 vocabulary scores were available, with the addition of L1 vocabulary to the model. For the baseline model, L1 vocabulary was allowed to covary with the other predictors, but regression paths of L1 vocabulary on syntactic integration and reading comprehension were not estimated. We then tested whether (a) adding a path from L1 vocabulary to syntactic integration and (b) adding a path from L1 vocabulary to reading comprehension improved model fit.

All path analyses were carried out using the maximum likelihood estimator in the lavaan package (version 0.5-20; Rosseel, 2012) in R (version 3.3.0; R Core Team, 2016). For all models, goodness of fit was assessed by the Chi square fit index (χ^2^), the comparative fit index (CFI), and the root mean square error of approximation (RMSEA). The χ^2^ fit index indicates amount of discrepancy between the hypothesized model and the data, meaning that a small and non-significant χ^2^ value indicates good model fit. RMSEA values below .05 and CFI values above .95 indicate good model fit. Differences between the fit of nested models were assessed by a χ^2^ difference test (Kline, 2011).

## Results

### Preliminary considerations

The data were screened for non-normality and outliers (defined as scores 3.29 *SD*s above or below the mean). Neither were detected. Five children (one monolingual, four bilingual) were absent during the classroom tests of syntactic integration and reading comprehension, resulting in missing scores on those variables. Four other children’s (one monolingual, three bilingual) inhibition scores were missing due to low accuracy on the Simon task. *t* tests and correlations were calculated using pairwise deletion of missing values. For the path analyses, missing data were handled with full information maximum likelihood estimation.

### Differences between L1 and L2 readers

We first investigated whether students speaking Dutch as a first (L1) or second (L2) language scored differently on language and cognitive skills (RQ1). Table 1 shows the descriptive statistics for all measures included in this study. Independent samples *t* tests showed that L2 readers were more efficient than L1 readers at word decoding (*t*(176) = − 2.99, *p* = .003, *d* = − .46) and pseudoword decoding (*t*(176) = − 3.77, *p* < .001, *d* = − .56), but that L2 readers scored lower than their L1 peers on vocabulary depth (*t*(176) = 4.10, *p* < .001, *d* = .62), vocabulary breadth (*t*(176) = 4.42, *p* < .001, *d* = .67), syntactic integration (*t*(171) = 2.04, *p* = .04, *d* = .31), and reading comprehension (*t*(171) = 3.10, *p* = .002, *d* = .47). No differences were found in working memory capacity (*t*(176) = .05, *p* = .96, *d* = .01) and inhibition (*t*(172) = − .99, *p* = .32, *d* = − .09).

**Table 1:** Mean scores on cognitive and linguistic measures for L1 and L2 children

	L1			L2		
	*n*	*M (SD)*	Range	*n*	*M (SD)*	Range
Home language vocabulary				56	42.5 (9.2)	13–59
Vocabulary depth	76	24.7 (7.0)	9–39	102	20.1 (7.6)	4–37
Vocabulary breadth	76	116.7 (10.2)	95–144	102	109.8 (10.5)	81–135
Word decoding	76	61.9 (11.3)	37–87	102	67.7 (13.8)	34–102
Pseudoword decoding	76	50.4 (14.1)	19–84	102	59.2 (16.4)	22–105
Syntactic integration	75	21.0 (6.7)	6–35	98	19.0 (6.0)	4–31
Working memory	76	4.3 (1.1)	2–7	102	4.3 (1.4)	1–8
Inhibition	75	−37 (46)	−148–55	99	−30 (43)	−164–71
Reading comprehension	75	25.6 (5.8)	9–36	98	23.0 (5.0)	7–33

### Predicting reading comprehension

To test whether the predictors of reading comprehension were different for L1 and L2 readers, we conducted multigroup path analyses. Table 2 shows the bivariate correlations between all variables as used in the subsequent path analysis. Correlations for the L1 group are shown below the diagonal, for the L2 group above the diagonal. For both groups, reading comprehension was positively correlated with vocabulary and syntactic integration. For the L1 group, working memory was also positively correlated with reading comprehension. In the L2 group, a positive correlation between reading comprehension and decoding was found.

**Table 2:**
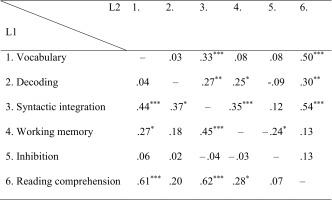
Correlations between variables used in the path analysis for L1 (*n* = 75–76) and L2 (*n* = 98–102)

Correlations for the L1 group are shown below the diagonal, for the L2 group above the diagonal

*** p < .001, ** p < .01, * p < .05

**Fig. 2:**
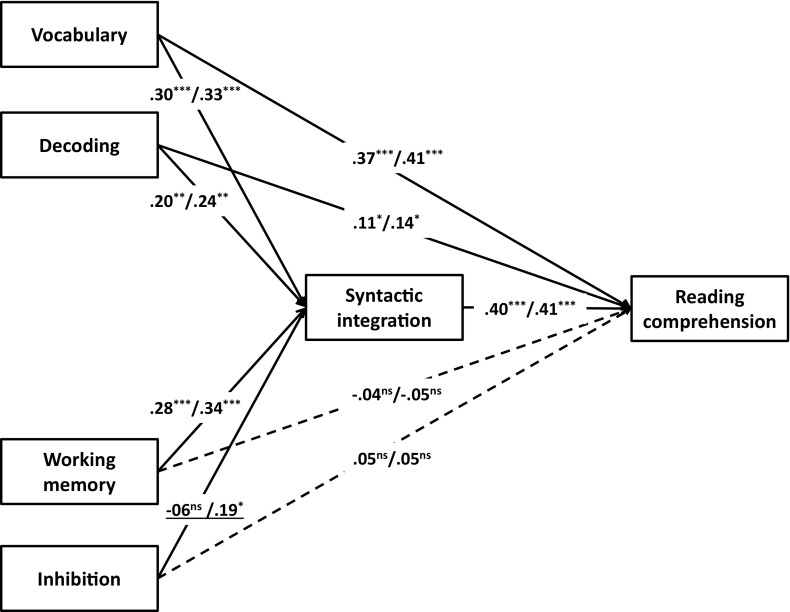
Model for predicting reading comprehension for L1 and L2 fourth grade readers. All unstandardized coefficients were constrained to be equal, except the path weight between inhibition and syntactic integration (underlined). Standardized coefficients are reported, for the L1 before and for the L2 group after the slash. All solid paths are significant, dashed paths are not. *** p < .001, ** p < .01, * p < .05. χ^2^(10, *N* = 178) = 9.39, *p* = .50, RMSEA = .00, CFI = 1.00; *R* ^*2*^_L1_ = .43, *R* ^*2*^_L2_ = .51

For the multigroup analysis, first a model in which regression coefficients were allowed to vary between groups was estimated. This model had good fit (χ^2^(2, *N* = 178) = .94, *p* = .63, RMSEA = .00, CFI = 1.00). We investigated whether the model could be simplified by constraining the unstandardized regression coefficients to be equal across groups. The only path for which an equality constrained marginally decreased model fit was the one between inhibition and syntactic integration (Δχ^2^ = 3.84, *p* = .05). For all other paths, an equality constraint led to a χ^2^ difference smaller than 2.5 (all *p*s > .10). For the final model, we thus constrained all paths except the one between inhibition and syntactic integration to be equal. The final model showed good fit (χ^2^(10, *N* = 178) = 9.39, *p* = .50, RMSEA = .00, CFI = 1.00). Figure 2 shows the final model’s standardized regression coefficients for the L1 and the L2 group. The model showed direct effects of decoding and vocabulary on reading comprehension, as well as indirect effects of decoding (β_L1_ = .60; β_L2_ = .65), vocabulary (β_L1_ = .77; β_L2_ = .82), and working memory (β_L1_ = .68; β_L2_ = .75) via syntactic integration on reading comprehension. The relation between inhibition and syntactic integration was significant for the L2 group only. The model explained 43% of the variance in reading comprehension for the L1 group and 51% for the L2 group.

### The role of L1 vocabulary in L2 reading comprehension

We then investigated the role of L1 vocabulary in L2 reading comprehension (RQ3). L1 vocabulary scores were available for a subgroup of 56 L2 children. L1 vocabulary was not significantly correlated with decoding, Dutch vocabulary, syntactic integration, working memory, and inhibition. There was a marginally significant correlation with reading comprehension (*r* = .26, *p* = .057). Spearman’s rank order correlation coefficient (ρ) showed that L1 vocabulary was significantly correlated with the amount of home language use with the mother (ρ = .26, *p* = .050), father (ρ = .35, *p* = .009), siblings (ρ = .27, *p* = .048), and extended family (ρ = .35, *p* = .007), but not with home language use with friends (ρ = .13, *p* = .333).

**Fig. 3:**
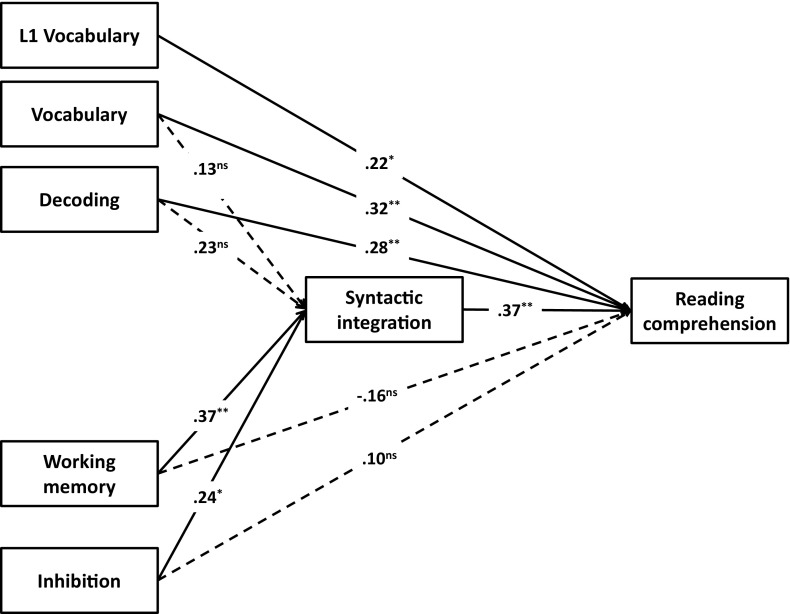
Model for predicting reading comprehension including L1 vocabulary for L2 fourth grade readers. Standardized coefficients are reported. All solid paths are significant, dashed paths are not. *** p < .001, ** p < .01, * p < .05. χ^2^(3, *N* = 57) = 2.67, *p* = .45, CFI = 1.00, RMSEA = .00; *R* ^*2*^ = .52

We first estimated the baseline model which included L1 vocabulary as an exogenous variable, but no regression paths between L1 vocabulary and other variables. This baseline model did not fit the data well (χ^2^(4, *N* = 57) = 7.86, *p* = .09, CFI = .92, RMSEA = .13). In subsequent models, we tested whether a path from L1 vocabulary to syntactic integration and a path from L1 vocabulary to reading comprehension improved model fit. This was only the case for the effect of L1 vocabulary on reading comprehension (Δχ^2^ = 5.18, *p* = .023). The path was thus retained in the final model. Figure 3 shows the standardized regression coefficients of the final model, which had good fit (χ^2^(3, *N* = 57) = 2.67, *p* = .45, CFI = 1.00, RMSEA = .00), and explained 52% of the variance in reading comprehension.

## Discussion

The present study extended previous research on differences between L1 and L2 reading comprehension, by looking into the central role of syntactic integration, by including executive control factors, and by taking home language proficiency into account. In line with earlier studies, we found that bilinguals decode faster than monolinguals, but perform worse on all other target language tasks. We found no differences between monolingual and bilingual children with regard to cognitive control tasks. The predictors of reading comprehension did not differ substantially between L1 and L2 readers. For both groups, lexical quality (decoding and vocabulary) predicted reading comprehension both indirectly via syntactic integration, and directly. Working memory indirectly predicted reading comprehension via syntactic integration for both groups. For L2 readers, inhibition also indirectly predicted reading comprehension via syntactic integration. In addition, for bilingual students, L1 vocabulary was found to have a positive effect on target language reading comprehension on top of the target language and executive control predictors.

Our first research question concerned mean differences on predictors of reading comprehension between bilingual and monolingual children. As expected, bilingual children had lower scores on Dutch vocabulary, syntactic integration, and reading comprehension tasks. This is consistent with a large body of research showing that bilingual children have weaker command of their L2 than monolingual children have of their first (and only) tongue (e.g., Babayiğit, 2014; Burgoyne et al., 2009; Lervåg & Aukrust, 2010; Leseman, 2000). This is most likely related to input quantity: A child acquiring two languages will normally have less input in each of them (for a review, see Unsworth, 2016). As Scheele, Leseman, and Mayo (2010) demonstrated, there is a trade-off between L1 and L2 input in the home environment of Turkish–Dutch and Moroccan–Dutch children. In addition, bilingual children’s L2 input is more likely to come from other non-native speakers, and this might impede vocabulary growth (Driessen, van der Slik, & de Bot, 2002; Hoff, Rumiche, Burridge, Ribot, & Welsh, 2014). Indeed, there are findings that suggest that English language input in the home environment does not improve English proficiency in Spanish–English bilingual children in the US (Hammer, Davison, Lawrence, & Miccio, 2009). Our data are consistent with these findings in that we found no relation between the amount of Dutch spoken in the home and Dutch vocabulary among bilingual children (amount of Dutch use with mother ρ = .15, *p* = .131; with father ρ = .13, *p* = .197; with siblings ρ = − .07, *p* = .484) .

Despite their weaker Dutch language skills, bilingual children were found to be faster decoders than their monolingual peers. This is consistent with earlier studies showing that bilinguals are as fast or faster than monolinguals at decoding (e.g., Babayiğit, 2015; Cremer & Schoonen, 2013; Droop & Verhoeven, 2003). Because of their smaller Dutch vocabularies, bilingual students encounter many unknown words when reading. We speculate that this strengthens the nonlexical route of reading (Coltheart, Rastle, Perry, Langdon, & Ziegler, 2001), resulting in fast decoding even of unknown words. Consistent with this account, the difference in decoding efficiency was more pronounced on the pseudoword task than on word reading.

In our sample, bilingual children did not differ from monolinguals in their inhibition or working memory. This is contrary to earlier evidence that there is a bilingual advantage in executive functions (e.g., Bobb, Wodniecka, & Kroll, 2013; Grundy & Timmer, 2017). Our findings add to the literature that contests these claims (De Bruin et al., 2015; Paap & Sawi, 2014). In the recent controversy surrounding the bilingual executive advantage, it has been suggested that the effect might be moderated by different factors, such as language dominance, socioeconomical status (SES), and cultural factors (for a discussion, see e.g., Paap, Johnson, & Sawi, 2015). As the bilingual children in our sample were all non-balanced bilinguals from lower-SES urban areas, it is beyond the scope of our study to clarify this issue further. For our purposes, it is most important to note that bilingual children in the current study did not differ from monolinguals in nonlinguistic cognitive tasks.

The second question of the current study was to what extent predictors of reading comprehension differed in strength between L1 and L2 readers. In line with reports by Babayiğit (2015) and Bowyer-Crane et al. (2016), we found no evidence for bilingualism moderating the effect of linguistic predictors of reading comprehension. Adding to the previous literature, we also investigated the hierarchical structure of linguistic predictors, and found that for monolingual and bilingual children, lexical quality (vocabulary and decoding) predicted reading comprehension both indirectly via syntactic integration and directly. The indirect effect supports the notion of the mental lexicon as a central input to sentence-level integration as posited by the Reading Systems Framework (Perfetti & Stafura, 2014). The direct effect might partly be attributed to the strong relationship between vocabulary and general world knowledge (Nagy & Herman, 1987). Our findings are then comparable to Oslund, Clemens, Simmons, Smith, and Simmons (2016). In a study with low-SES middle school students, they found that vocabulary and background knowledge were highly related, but that vocabulary contributed to reading comprehension indirectly via sentence comprehension and inferencing, while background knowledge was an independent predictor of reading comprehension.

Next to linguistic predictors, we also investigated the role of executive functions. The effect of working memory on reading comprehension was fully mediated by syntactic integration. Working memory has long been linked to both syntactic processing (e.g., Caplan & Waters, 1999; King & Just, 1991) and reading comprehension (e.g., Cain, 2006; Sesma et al., 2009), but syntactic integration as a link between working memory and comprehension is seldom made explicit. Reading comprehension problems linked to working memory are more often investigated through the lens of comprehension monitoring or inferencing (e.g., Cain, Lemmon, & Oakhill, 2004; Currie & Cain, 2015). Monitoring and inferencing tasks resemble syntax tasks in that all require unification of smaller elements into larger units of meaning. In addition, monitoring and inferencing tasks often rely on syntactic processing (e.g., pronoun resolution).

While the effects of lexical quality and working memory on reading comprehension were the same for monolinguals and bilinguals, only bilinguals showed a significant effect of inhibition on syntactic integration. As the interaction effect was only marginally significant, it needs to be replicated and interpreted with caution. Nevertheless, it is consistent with recent neuroimaging studies: In a meta-analysis of the neural representation of L1 and L2, Sebastian et al. (2011) noted that less-proficient bilinguals showed less activation than more proficient L2 speakers in brain areas involved in semantic processing, and more in areas involved with cognitive control when performing L2 tasks. Prehn et al. (2017) also found that L2 speakers recruited executive control areas more than L1 speakers when performing a grammaticality judgement task. This is consistent with our tentative conclusion: For speakers with weaker semantic representations, syntactic integration is more effortful and requires attentional processing.

Our third research question concerned the impact of L1 vocabulary on L2 reading. We found that L1 vocabulary had a positive effect on Dutch reading comprehension on top of the predictors discussed above. This strengthens earlier findings of a positive influence of L1 proficiency on L2 achievement (Lervåg & Aukrust, 2010; Proctor et al., 2006), even after controlling for nonlinguistic predictors. There are several possible—complementary—explanations for this finding. L1 vocabulary might be an indicator of general language aptitude and a language-rich environment, both of which are conductive to reading development. A well-developed L1 vocabulary might also help to scaffold L2 acquisition. For concepts that are already known in the L1, only a new label has to be learned and fine-tuned in the L2. In line with this reasoning, Scheele et al. (2010) found that L1 vocabulary positively predicted L2 vocabulary in young children. In our sample, however, L1 vocabulary and L2 vocabulary did not correlate. This might be the result of a trade-off: L1 vocabulary might help L2 vocabulary development, but it requires time to build. Time spent with L1 input is time not spent learning the L2. As quality of input is possibly higher in the L1, however, this is not a reason to discourage L1 input. L1 vocabulary can be seen as an indicator of conceptual richness and amount of world knowledge. This is consistent with our finding that L1 vocabulary directly predicts L2 reading comprehension, without an indirect effect via syntactic integration.

At this point, several limitations of this study must be acknowledged. We did not collect multiple measures of all constructs and test enough participants for latent-variable modeling. This introduces a larger measurement error in the models. However, it allowed us to test a broader range of both cognitive and linguistic predictors. In addition, the group that could be tested on L1 vocabulary was relatively small, due to the composition of the sample. Because all children in all classrooms were tested, the bilingual students had diverse language backgrounds. This improved representativeness, but made it very difficult to collect L1 data for all bilingual students. Future studies on the role of L1 knowledge in L2 reading comprehension are necessary, and should take variation in language background into account. Due to the diverse language background of the participants, we also did not assess working memory and syntactic integration in the bilingual children’s L1. However, Dutch was the language of schooling for all participants, and previous research found digit span performance to be equal in the L1 and L2 of children whose L2 was their school language (Chincotta & Underwood, 1996). Concerning syntactic integration, it seems that L1 vocabulary is a more plausible precursor of L2 reading comprehension than L1 syntactic integration. Vocabulary pertains to conceptual richness, which is useful for both languages, whereas knowledge of syntax would transfer less well between languages. Further research is necessary to test this assumption. Lastly, our results describe a single point in time and we therefore cannot make any claims about causality. Our findings need to be confirmed by longitudinal data.

The main educational implication of our findings is that target language lexical quality and working memory are of crucial importance, and serve as input to higher-level processing, which should be supported. For bilinguals, L1 vocabulary also has a positive influence on L2 reading comprehension, and home language use should be encouraged while L2 proficiency is built.

To conclude, the present study shows that bilingual children in the Netherlands still lag behind their monolingual peers in reading comprehension, after almost 4 years of Dutch reading instruction. This is a consequence of lower L2 proficiency, rather than of decoding ability or cognitive factors. Reading comprehension is a result of similar processes in L1 and L2 readers. For both, lexical quality predicts reading comprehension directly and indirectly via syntactic integration. Executive control also has an indirect effect on reading comprehension via syntactic integration. This is most apparent for working memory, which plays a role for both bilinguals and monolinguals. The role of inhibition seems to be stronger for bilinguals, possibly as a compensatory mechanism for lower lexical quality. In addition, L2 reading comprehension is positively influenced by L1 vocabulary. Together, our results show that reading comprehension is a result of similar processes in L1 and L2 readers, with a central role for sentence-level processing. L2 readers might require more executive control for syntactic integration, and rely on their total language resources to arrive at successful comprehension.
